# Socio-demographic, dietary, exercise, and mental health factors associated with food addiction symptoms in university students

**DOI:** 10.1186/s41155-025-00363-0

**Published:** 2025-10-30

**Authors:** Iasmim Cristiane de Alcântara, Bruna Eugênia Ferreira Mota, Agatha Kelly da Luz Castro, Gabriela Guerra Leal Souza

**Affiliations:** 1 Laboratory of Psychophysiology, Institute of Exact and Biological Sciences, Department of Biological Sciences, Federal University of Ouro Preto, Ouro Preto, Brazil; 2 Graduate Program in Health and Nutrition, Federal University of Ouro Preto, Ouro Preto, Brazil; 3 National Institute of Science and Technology in Social and Affective Neuroscience, INCT-SANI, CNPq, São Paulo, Brazil; 4 Social and Cognitive Neuroscience Laboratory, Mackenzie Presbyterian University, São Paulo, Brazil

**Keywords:** Food addiction, Ultra-processed food, University students, Mental health, Socio-demographic factors

## Abstract

**Introduction:**

Food addiction is characterized by repeated and uncontrolled consumption of food, usually ultra-processed foods.

**Objective:**

To investigate the impact of sociodemographic, health, lifestyle, and dietary factors on food addiction symptoms.

**Methods:**

A total of 512 university students (both sexes, aged between 18 and 35 years) provided information on completion that included the variables include family income, physical activity, psychiatric and physical disorders, and alcohol, drug, and tobacco use. They also shared dietary information such as main meal types and whether they added salt to food. Food addiction symptoms were assessed using the Yale Food Addiction Scale 2.0. We used a multiple linear regression model was used to investigate predicted changes in the number of food addiction symptoms.

**Results:**

The model hadshowed an adjusted R^2^ adjusted of 0.167 (and *p* < 0.001). Female sex (*B* = 0.506; *p* = < 0.,0401), low family income (from low to moderate: *B* = -0.803, *p* = 0.,002; and from low to high: *B* = -0.732, *p* = 0.024), physical (*p* = 0,046) and psychiatric disorders (*B* = 1.062, *p* < = 0.001,08), practice of physical activity (*B* = -0.682, *p* = 0.009) and be in current dieting (*B* = 1.498, *p* < 0.001) predicted morea use of tobacco and derivatives (*p* = 0,020) food addiction symptoms. (*p* = 0,018) (*p* = 0,025) consumes less than five meals a day (*p* < 0,001).

**Conclusion:**

Food addiction symptoms were found to be a multifactorial phenomenon, associated with sociodemographic and economic status, diet, exercise, and mental health. The limitations of this study of this study includewere its cross-sectional design, lack ofno dietary consumption data, sample convenience sampling-based, self-selection bias, and simplification ed collection of dietary concerns.

## Introduction

Food addiction refers to the phenomenon in which consumers have a dependency relationship with certains, characterized by a pattern of repeated and uncontrolled food consumption (Kalon et al., 2016). It is well-established that this addiction is strongly associated with ultra-processed foods that contain large quantities of sugar, fat, and additives designed to maximize their appeal (Curtis & Davis, 2014; David et al., 2018; Lemos et al., 2022; Schulte et al., 2015). Understanding this relationship is fundamental because the root of food addiction may be linked to the brain’s reward system.

This reward system involves structures such as the nucleus accumbens and dopaminergic system, which is responsible for the release of the “happiness hormone” dopamine (Lindgren et al., 2018; Romer et al., 2019). Related to the generation of pleasure, the consumption of highly palatable foods, such as ultra-processed foods, can hyperactivate this reward system (Davis et al., 2013). With the frequent consumption of such foods, a phenomenon known as tolerance might occur. Increasingly larger stimuli may be required to trigger the desired effect, that is, pleasure, which is one of the key elements in the definition of addiction (Lustig, 2020). Stimuli from ultra-processed foods and drugs have been found to activate similar brain areas, including the striatum, amygdala, orbitofrontal cortex, and anterior insula. This highlights the overlap of the underlying neurobiological mechanisms between these two types of addiction (Tang et al., 2012). Thus, food addiction shares attributes with substance use dependence, including cravings, withdrawal, and tolerance (Gearhardt & Schulte, 2021).

Despite that, food addiction is not officially recognized as a disorder by the Diagnostic and Statistical Manual of Mental Disorders (DSM-5) (American Psychiatric Association, 2014). The Yale Food Addiction Scale (YFAS 2.0) consists of a series of questions based on the diagnostic criteria established by the DSM-5 for substance use dependence, adapted for the context of food consumption dependence. The tool allows for the assessment of addictive eating behaviors, such as loss of control over consumption, persistence in use despite adverse consequences, and unsuccessful attempts to reduce consumption despite the desire to do so. The authors of this instrument suggested that it facilitated quantifying symptoms and determining the severity of food addiction (Gearhardt et al., 2016).

The modern food environment, characterized by the high availability and aggressive marketing of ultra-processed foods (Saúde, 2015), may amplify individual vulnerabilities to food addiction.In this context, beyond the neurobiological basis, is (LaFata & Gearhardt, 2022). In this context, beyond the neurobiological basis, it is this crucial to consider the role of individual differences in the modulating susceptibility to addictive eating behaviors.

A growing body of evidence indicates that factors such as age, sex, body mass index (BMI), income, and sleep quality are important determinants of food addiction (dos Santos et al., 2020; Romero-Blanco et al., 2021; Schulte & Gearhardt, 2018). Additionally, the presence of psychological disorders, such as depression and anxiety, may increase the predisposition to these symptoms (Borisenkov et al., 2018). A lack of control over food consumption can have serious consequences on physical and mental health (Vasiliu, 2021). Hence, it is making it essential to identify the most vulnerable groups.

Among these vulnerable groups, university students deserve special attention. This population experiences is going through a transitional phase marked by greater autonomy, changes in eating patterns, exposure to academic stress, use of recreational substances, and a higher prevalence of depressive symptoms. These are all factors that can increase the risk of developing dysfunctional eating behaviors (Romero-Blanco et al., 2021). Despite the growing research interest in this topic, there are still gaps in the literature regarding the profiles of Brazilian university students with the most symptoms of food addiction symptoms are poorly described., In particular, there is a gap especially concerning with regard to the associations between these symptoms and with sociodemographic, health, lifestyle, and dietary eating habits factors, with only one study on Brazilian university students (Romero-Blanco et al., 2021). Identifying these individuals will enable targeted preventive and therapeutic actions, thereby promoting the health and wellbeing of the academic community.

Thus, this study compared the number of food addiction symptoms across a variety of individual characteristics, such as sociodemographic factors, health, lifestyle, and dietary habits, in a sample of Brazilian university students. The goal was to profile university students according to food addiction.

## Methods

### Participants and study design

All college students from Federal University of Ouro Preto (UFOP) were invited to participate in this cross-sectional study via an institutional email. Sent by the corresponding sectors of the institutions, this email contained a brief explanation of the study and a link to the questionnaire on Google Forms (Google®). The data were collected between May 2022 and August 2023. Before data collection that, we did a pilot test was conducted of the questionnaires with ten10 participants to guarantee that all questions were understandable and that there were no operationalization problems. To assess the representativeness of the sample in relation to the total population of UFOP students (*N* = 11,858), the sample size was calculated using the formula for estimating population proportions with, considering a 5% margin of error, a 95% confidence level (Z = 1.96), and an expected proportion of 50% (*p* = 0.5). The finite population correction was applied, as recommended by Levine, Berenson, and Stephan (2000). The minimum required sample size required was estimated to be 373approximately 373 students.

For inclusion in the study, the inclusion criteria were to be required to be a university/college students aged and be between 18 and 35 years old. There were no exclusion criteria for this study. Invitations were sent to 11,858 university students; 582 of them responded to the questionnaire. Thirty-eight students were excluded because of duplicate responses. The inclusion criteria were to be a college student and be between 18 and 35 years old. There was no exclusion criteria. We did not have incomplete questionnaires. The final sample comprised 512 university students. Participants did not receive compensation for their participation.

#### Questionnaires

##### Sociodemographic factors and health

The participants answered questions about their sex (female or male); age (in years); ethnicity/race (white, brown, black, yellow, indigenous, undeclared, or others); monthly family income in minimal wages (less than 2, between 2 and 5, or more than 5, in the Brazilian currency, real, R$); history of physical or mental disorders (yes or no); regular physical exercise (duration and type); and use of alcohol, tobacco, and other recreational drugs (yes or no for all).

##### Modified Yale Food Addiction Scale 2.0 (mYFAS2.0)

Food addiction symptoms were measured using the modified Yale Food Addiction (mYFAS 2.0) (Schulte & Gearhardt, 2017), which is cross-culturally validated for the Portuguese language (Nunes-Neto et al., 2018a). It is a self-report scale designed to measure addictive eating behaviors. It consists of 13 items: 11 items address symptoms and 2 items assess the clinical significance of those symptoms (items 5 and 6), indicating the presence of significant distress or impairment related to food consumption. Participants reported the frequency of addictive eating behaviors over the past 12 months, ranging from never to every day. To establish the symptom count score, all the scores for the 11 symptom-related questions were summed. The alfa de Cronbach’s alpha for our sample was 0.878, demonstrating high reliability.

##### Dietary variables

Participants were asked about particular eating practices. Participants’ concerns about with their diets was assessed using by the question, “Are you currently concerned about your diet?” with response options “no,” “yes,” and “unable to answer”. Frequency of food label reading was assessed by the question, “How often do you read the nutrition facts label and/or ingredient list on food packages?” with response options “never,” “almost never,” “sometimes,” “nearly always,” and “always.” Number of meals per day was assessed by asking, “How many meals (breakfast, snacks, lunch, dinner) do you usually eat per day?” with numeric responses ranging from “1” to “6 or more”. For analytical purposes, responses were categorized as “less than 3”, “between 3 and 5,” and “more than 5”.

The type of food consumed at the main daily meal (i.e., the largest meal of the day) was assessed with the question, “What type of food do you usually consume in your main meal on a typical day?” with response the options: “campus restaurant,” “homemade meals,” “fast food,” and “restaurant and similar (non fastnon-fast food)”. Homemade meals were defined as those prepared in the participants'own homes. This distinguishes them from restaurant meals, where the preparation process is unknown to the participant, and they have no control over the ingredients used, cooking methods, or other aspects of the meal. By note that by evaluating the usual types of meals consumed, we intended to evaluate whether meals were consumed outside the participants’ homes (IBGE, 2020). Participants were also asked whether they usually added salt to their meals (“no” or “yes”), whether they had ever followed a weight loss diet in the past (“no” or “yes”), and whether they were currently following any type of diet at the time of completing the questionnaire (“no” or “yes”).

### Statistical analysis

The database was constructed using Microsoft Office Excel 2013^©^ software, and statistical tests were performed using Jamovi software version 2.6.44. Categorical variables are presented as absolute numbers (n) and percentages. Since the normality of quantitative data was tested using the Kolmogorov–Smirnov test and presented non-normal distributions, these data were presented using the median, 25th percentile, and 75th percentile (p25 and p75, respectively).

Spearman’s correlation was used to explore the association between Pearson food addiction symptom counts and all variables. If the variable was significantly associated with food addiction symptom counts, it was included in the multiple linear regression model. In this model, the dependent variable was the food addiction symptom counts., The independent variables were monthly family income, sex, practice of physical activity, diagnosis of physical disease, diagnosis of psychiatric disorders, ease, tobacco use, salt added to meals, and being currently on a diet.

The significance level was set at *p* < 0.05.

### Ethics declarations

This study was conducted in accordance with the rigid principles of the Declaration of Helsinki and its amendments. All procedures were approved by the Research Ethics Committee of the Federal University of Ouro Preto (CAAE: 31,544,220.2.0000.5150). All the participants provided informed consent before participating in the study. The data will be made available upon reasonable request.

## Results

The participants’ ages ranged from 18 to 35 years (median = 23.,00 years, p25 = 21.,00 years, and p75 = 28.,00 years). See Table 1 for descriptive statistics on sociodemographic factors.

**Table 1:** Socio-demographic factors, health, and lifestyle characteristics of the participants (*n* = 512)

Variables	*n*	%
Sex		
Male	164	67.97
Female	348	32.03
Ethnicity/Race		
White	255	49.80
Non-white	169	33.01
Black	79	15.43
Yellow	4	0.78
Indigenous	2	0.39
Mixed-race	2	0.39
Undeclared	1	0.20
Monthly family income (in minimal wages)^a^		
Less than 2	238	46.48
Between 2 and 5	181	35.36
More than 5	93	18.16
Diagnosis of physical disease		
No	452	88.28
Yes	60	11.72
Diagnosis of psychiatric disease		
No	349	68.16
Yes	163	31.84
Practice physical activity^b^		
Active	137	26.76
Inactive	375	73.74
Use of tobacco and derivatives		
No	424	82.81
Yes	88	17.19
Alcohol consumption		
No	165	32.23
Yes	347	67.77
Other recreational drug use		
No	419	81.84
Yes	93	18.16

^a^The minimum wage in force in 2022 in Brazil (R$ 1,312.00) was taken as a reference

^b^Physically active individuals met the World Health Organization recommendation of 150 to 300 min of moderate to vigorous aerobic activity per week

The characterization of the sample in terms of dietary variables is described in Table 2.

**Table 2:** Description of participants regarding dietary variables (*N* = 512)

Dietary variables	*N*	%
Concerned about their diet		
No	58	11.33
Yes	423	82.,62
Unable to answer	31	06.05
Frequency of label reading		
Never	77	15.04
Almost never	113	22.07
Sometimes	111	21.68
Nearly always	118	23.05
Always	93	18.16
Number of meals per day		
Less than 3	50	9.77
Between 2 and 5	491	86.13
More than 5	23	4.10
Main meal		
Campus restaurant	206	40.23
Homemade	255	49.81
Fast food	20	3.91
Restaurants and similar (non-fast food)	31	6.05
Salt added to meals		
No	290	56.64
Yes	222	43.36
Being on a diet		
No	63	12.31
Yes	449	87.69
Ever been on a diet to lose weight		
No	306	59.77
Yes	206	40.23

The study obtained median, p25, p50 and p75 of the food addiction questionnaire (mYFAS2.0) was 0.00; 0.00; 0.00 and 2.00, respectively, for the food addiction questionnaire (mYFAS2.0). Which indicates that most of the majority of participants showed few symptoms. However, a portion of the sample reported higher levels of food addiction symptoms, highlighting the relevance of exploring associated factors.

The correlation analysis showed that food addiction symptoms were positively associated with physical and psychiatric disease (Rho = 0.88, *p* = 0.046; and Rho = 0.28, *p* < 0.001, respectively), tobacco consumption (Rho = 0.10, *p* = 0.02), salt added to meals (Rho = 0.10, *p* = 0.03) and be in currently being on a diet (Rho = 0.27, *p* < 0.001). Furthermore, these symptoms were, and negatively associated with male sex (Rho = -0.19, *p* < 0.001), monthly family income (Rho = -0.13, *p* = 0.003) and practice of exercise (Rho = -0.12, *p* = 0.008). See Table 3 (for all correlation coefficients Rho and *p* values details see Table 3). All these variables were included in a multiple linear regression model to determine show which variables predicted the increase in food addiction symptoms. The model has shown *R*^*2*^ = 0.181, adjusted *R*^*2*^ adjusted = 0.167, and *p* < 0.001 in the F- test. The results showed that being female, having a lower less monthly family income, having a diagnosis of psychiatric disorders, being on a current diet, and non-practicing physical activity predicted more food addiction symptoms (for details see Table 4 for more details), suggesting a greater susceptibility of women to addictive eating behavior. Similarly, indicating a possible influence of the socioeconomic context on food addiction and/or physical, which may reflect an association between mental health conditions and the severity of dysfunctional eating behavior and those who reported not using tobacco or related products (Table 3), suggesting that meal regularity may be associated with greater food control, indicating that food quality can also influence food addiction.

**Table 3:** Matrix of spearman’s correlations

		**Symptom count for food addiction**	**Monthly family income**	**Ethnicity/race**	**Sex**	**Physical activity**	**Physical disease**	**Psychiatric disorder**	**Tobacco use**	**Alcohol use**	**Recreational Drug use**	**Concerned about diet**	**Label reading**	**M**
Symptom count for food addiction	**Rho**	__												
	**gl**	__												
	***p*-value**	__												
Monthly family Incomeª	**Rho**	−0.130	__											
	**gl**	510	__											
	***p*-value**	0.003	__											
Ethnicity/race	**Rho**	0.027	−0.162	__										
	**gl**	510	510	__										
	***p*-value**	0.513	<.001	__										
Sex	**Rho**	−0.189	0.077	−0.029	—									
	**gl**	510	510	510	—									
	***p*-value**	<.001	0.080	0.509	—									
Practice physical activity^b^	**Rho**	−0.017	0.100	−0.003	0.067	__								
	**gl**	510	510	510	510	__								
	***p*-value**	0.008	0.024	0.942	0.128	__								
Diagnosis of physical disease	**Rho**	0.088	−0.041	−0.030	−0.068	−0.056	__							
	**gl**	510	510	510	510	510	__							
	***p*-value**	0.046	0.359	0.499	0.125	0.209	—							
Diagnosis of psychiatric disease	**Rho**	0.282	−0.028	−0.106	−0.137	−0.082	0.207	__						
	**gl**	510	510	510	510	510	510	__						
	***p*-value**	<.001	0.533	0.016	0.002	0.065	<.001	__						
Use of tobacco and derivatives	**Rho**	0.103	−0.069	−0.086	0.042	−0.065	0.092	0.044	__					
	**gl**	510	510	510	510	510	510	510	__					
	***p*-value**	0.020	0.118	0.051	0.339	0.143	0.038	0.317	__					
Alcohol consumption	**Rho**	0.071	−0.035	−0.111	−0.055	−0.084	0.030	0.005	0.248	__				
	**gl**	510	510	510	510	510	510	510	510	__				
	***p*-value**	0.108	0.432	0.012	0.214	0.059	0.493	0.915	< 0.001	__				
Other recreational drug use	**Rho**	0.047	−0.067	−0.102	0.100	−0.056	0.033	0.015	0.403	0.249	__			
	**gl**	510	510	510	510	510	510	510	510	510	__			
	***p*-value**	0.284	0.127	0.021	0.024	0.207	0.455	0.732	<.001	< 0.001	__			
Concerned about their diet	**Rho**	0.056	−0.009	0.071	−0.086	0.039	0.002	0.006	0.017	0.004	−0.040	__		
	**gl**	510	510	510	510	510	510	510	510	510	510	__		
	***p*-value**	0.203	0.837	0.107	0.052	0.379	0.961	0.901	0.703	0.935	0.370	__		
Frequency of label reading	**Rho**	0.073	0.006	−0.076	−0.081	0.214	0.073	0.133	−0.044	−0.006	−0.017	0.063	__	
	**gl**	510	510	510	510	510	510	510	510	510	510	510	__	
	***p*-value**	0.101	0.892	0.087	0.067	<.001	0.098	0.003	0.325	0.886	0.699	0.154	__	
Number of meals per day	**Rho**	−0.049	0.074	0.075	−0.056	0.106	−0.059	−0.044	−0.086	−0.036	−0.051	0.087	0.111	
	**gl**	510	510	510	510	510	510	510	510	510	510	510	510	
	***p*-value**	0.226	0.095	0.091	0.205	0.016	0.182	0.321	0.053	0.410	0.251	0.049	0.012	
Main meal	**Rho**	0.033	0.022	−0.036	0.010	−0.074	0.001	0.066	−0.034	−0.051	−0.024	−0.017	−0.011	α
	**gl**	510	510	510	510	510	510	510	510	510	510	510	510	
	***p*-value**	0.457	0.618	0.412	0.815	0.094	0.977	0.138	0.440	0.250	0.590	0.694	0.807	α
Salt added to meals	**Rho**	0.099	−0.024	0.002	−0.153	−0.057	0.049	0.028	0.051	0.072	0.078	−0.003	−0.022	α
	**gl**	510	510	510	510	510	510	510	510	510	510	510	510	
	***p*-value**	0.025	0.590	0.962	<.001	0.198	0.270	0.525	0.253	0.103	0.076	0.953	0.616	α
Being on diet	**Rho**	0.273	0.021	0.005	−0.136	0.107	0.035	0.183	−0.047	0.003	−0.077	0.090	0.252	α
	**gl**	510	510	510	510	510	510	510	510	510	510	510	510	
	***p*-value**	<.001	0.634	0.907	0.002	0.016	0.424	<.001	0.293	0.942	0.083	0.043	<.001	α
Ever been on a diet to lose weight	**Rho**	0.073	0.133	−0.062	0.087	0.257	0.011	−0.039	−0.013	−0.009	−0.099	0.066	0.213	α
	**gl**	510	510	510	510	510	510	510	510	510	510	510	510	
	***p*-value**	0.100	0.003	0.162	0.049	<.001	0.797	0.378	0.768	0.841	0.025	0.137	<.001	α
Age	**Rho**	−0.038	−0.086	0.090	0.057	0.066	−0.022	−0.014	0.026	−0.090	0.044	−0.013	0.193	α
	**gl**	510	510	510	510	510	510	510	510	510	510	510	510	
	***p*-value**	0.388	0.052	0.043	0.196	0.137	0.955	0.758	0.566	0.043	0.319	0.762	<.001	α

^a^The minimum wage in force in 2022 in Brazil (R$ 1,312.00) was taken as a reference

^b^Physically active individuals met the World Health Organization recommendation of 150 to 300 min of moderate to vigorous aerobic activity per week

**Table 4:** Multilinear Regression model: – symptom count for food addiction

Predictor	Estimates	Standard error	*t*	*p*
Interceptoª	0.909	0.279	3.258	0.001
Monthly family income				
2–1	−0.803	0.253	−3.182	0.002
3–1	−0.732	0.311	−2.353	0.019
Sex				
1–2	0.506	0.248	2.039	0.042
Practice physical activity				
1–0	−0.682	0.258	−2.641	0.009
Diagnosis of physical disease				
1–0	0.235	0.357	0.657	0.511
Diagnosis of psychiatric disorderease				
1–0	1.062	0.252	4.217	<.001
Tobacco consumption				
1–0	0.104	0.300	0.348	0.728
Salt added to meals				
1–0	0.156	0.230	0.679	0.497
Being currently on a diet				
1–0	1.498	0.236	6.349	<.001

ª Represents the reference level

This absence of association suggests that these factors were not determinants for food dependency in this sample, unlike the other variables presented.

Taken together, in an integrative way, the results of this study indicate that food addiction symptoms are predicted by sociodemographic factors (such as sex and income), diagnosis of psychiatric disorders, ease, current being on a diet, and practice of physical activity. On the other hand, the other sociodemographic variables, use of alcohol, tobacco, and drugs, physical diseases, and predicted more food addiction symptoms. Similarly associated with sociodemographic factors (such as sex and income), mental and physical health conditions, and eating behavior patterns, while other variables, such as race/ethnicity, substance use and diet concerns, were not significantly related to symptom severity.

## Discussion

This study investigated the differences in the number of food addiction symptoms according to the sociodemographic, health, lifestyle, and dietary characteristics of college students. Female sex was a predictor of a higher median number of symptoms than male sex. This result corroborates a previous systematic review indicating a higher prevalence of food addiction among females (12.2%) (Pursey et al., 2014). This was also the case for a Brazilian non-clinical sample in which females tended to have higher symptom counts than males (Nunes-Neto et al., 2018b). However, there is conflicting information in the literature regarding sex differences in food addiction.

A meta-analysis showed a higher prevalence of food addiction in males (27%) compared to 24% in females (Praxedes et al., 2022). Although food addiction is not included in the DSM-5, its symptoms among females may be attributed to various factors such as hormonal fluctuations, emotional eating behaviors, and societal pressures related to body image and dieting (Contessoto et al., 2021); Fattore et al., 2014). Eating disorders, in general, are considered more prevalent in this group (Qian et al., 2013). Studies have shown that women tend to use food as an emotional coping mechanism (Hussenoeder et al., 2022; Rosenqvist et al., 2022), which may have contributed to the higher number of food addiction symptoms observed in this study.

Emotional eating behavior is often associated with the consumption of ultra-processed foods designed to activate the brain’s reward centers (Davis et al., 2013). As this study did not directly assess the relationship between emotional eating and the consumption of ultra-processed foods, future research should explore this association thoroughly. This could provide a clearer understanding of the observed sex differences in food addiction.

Participants diagnosed with psychiatric disorders exhibited more food addiction symptoms than those without psychiatric disorders. This may be explained by the interconnection between brain reward systems, which are often dysregulated in psychiatric conditions, and addictive behaviors (Grajek et al., 2022). Individuals with psychiatric disorders such as depression and anxiety may also report excessive food consumption, particularly foods high in sugar and fat, as a form of “self-medication” to alleviate negative emotional symptoms (Burrows et al., 2018; Konttinen et al., 2019; Stariolo et al., 2024). This may also explain why female participants exhibited more symptoms, as this group had a high prevalence of psychological disorders, which may have increased their vulnerability to food-related issues (Altemus et al., 2014). A cross-sectional study based on data from an anonymous Brazilian online anonymous survey platform reportedly found a higher prevalence of food addiction among women (Nunes-Neto et al., 2018b). Thise condition was associated with depressive symptoms, bipolar disorder, excoriation disorder, experiences of psychological and sexual abuse in childhood, and as well as reduced quality of life in the physical, psychological, social, and environmental domains (Nunes-Nneto et al., 2018b).

This study showed that physically active participants, according to the World Health Organization (WHO) recommendations, exhibited fewer food addiction symptoms than those with sedentary behaviors. These findings are consistent with those of previous studies (Romero-Blanco et al., 2021; Zielińska et al., 2024), which also reported observed a higher prevalence of symptoms among sedentary individuals. Additionally, research indicates that individuals with addictive behaviors tend to spend less time engaging in physical activity and more time engaging in sedentary behaviors (Li et al., 2018). Our results support the hypothesis that regular physical activity may serve as a protective factor against dysfunctional eating behaviors. These findings highlight the importance of promoting physical activity as a potential strategy for the prevention and management of food addiction, while also suggesting the need for further research to clarify the mechanisms involved in this relationship in the assessment of particularly participants’ estimations for future studies.

Difficulty in accessing adequate and balanced nutrition is common among low-income groups. This often leads to higher consumption of ultra-processed foods (Louzada et al., 2023), which are convenient, low-cost, and long-lasting, but have a nutritionally imbalanced composition (Monteiro et al., 2010). This may explain why participants with a lower monthly family income exhibited more symptoms of food addiction than those with a higher family income.

In this study, individuals who reported being on following a diet exhibited a higher number of food addiction symptoms. This result supports the notion that attempts to control weight, especially through restrictive approaches, may contribute to the development of food addiction (Brownell & Walsh, 2017). Restrictive diets can generate a sense of deprivation, intensify cravings for certain foods, and lead to episodes of loss of control (Meule, 2020; Polivy et al., 2005; Wardle, 1987). In addition, these diets may interfere with the body’s natural hunger and satiety signals, encouraging irregular eating patterns similar to those observed in addictive behaviors (Whatnall et al., 2022). Rather than preventing disordered eating, strict dietary control may exacerbate it, in some cases, exacerbate it.

Based on these results found, it is possible to suggest the implementation of specific interventions aimed at university students with greater vulnerability to food addiction, particularly women, low-income individuals, and those with psychiatric disorders. Multidisciplinary programs that include nutritional education, mental health support, and strategies for promoting healthy eating habits can be effective in reducing addictive eating behaviors. In addition, institutional policies that increase the availability of healthy foods in universities and limit exposure to ultra-processed foods may can contribute to a more protective food environment. Interventions must need to be sensitive to students’ socioeconomic conditions, and also considering the roles of emotions and stress in their relationships with food.

This present study contributes to the Literature on food addiction by demonstrating an inverse association with the practice of physical activity, indicating that behaviors related to health promotion may have a protective effect against the symptoms of food addiction. Furthermore, our findings reinforce the importance of considering both emotional and socioeconomic factors, given that food addiction is a complex and multifactorial phenomenon. This study aimed to provide a broader understanding of how everyday practices relate to the risk of food addictionFA byBy including diverse dietary and Lifestyle habits, such as meal frequency, food preparation methods, and salt use, this study provides a more comprehensive understanding of how everyday practices relate to the risk of food addiction this study aimed to provide a broader understanding of how everyday practices relate to the risk of food addiction. Although most of these variables were not significantly associated with the outcome, being on a diet was linked to a higher number of symptoms, highlighting the important relevance of considering how weight control strategies may contribute to dysfunctional eating behaviors. Finally, this study it adds to the limited body of Brazilian research on food addiction in non-clinical, young adult populations, offering insights that can inform culturally relevant public health strategies within university settings. The majority of Brazilian and Latin American studies use the Modified Yale Food Addiction Scale 2.0 to determine if the participant has or has not the “food addiction disease”, but here, as was used by Romero-Blanco et al. (2021), we used this questionnaire to count the symptoms of food addiction.

This study had some limitations. First, its cross-sectional design did not allow causal relationships to be established. Second, the study potentially underestimated food addiction symptoms due to the self-reported nature of the data. Third, no dietary consumption data were collected for this study, which restricts quantitative and qualitative inferences regarding the adequacy of students’ food consumption. Fourth, because the sample was convenience-based, the results cannot be generalized to the broader Brazilian population. Fifth, the sampling procedure was based on voluntary participation through email invitations. This, which may have introduced self-selection bias. Sixth, given the complexity of dietary concerns as a construct, the question format with limited response options may not fully capture the different dimensions or severities of dietary concerns among participants.

## Conclusion

This study identified factors associated with the number of food addiction symptoms among Brazilian university students. More higher number of symptoms were as observed among women, individuals with psychiatric disorders, those with lower family income, and participants who reported being on a diet. These findings reinforce the importance of considering emotional and socioeconomic factors when addressing food addiction in young adults. Notably, regular physical activity was inversely associated with food addiction symptoms, suggesting a potential protective effect. Although age was not significantly associated with the outcome in this sample, it remains a relevant demographic factor that should be considered in future studies, particularly in relation to lifestyle transitions experienced during university life.

Overall, the results highlight the need for targeted, multidisciplinary interventions within university settings, especially for the most vulnerable groups. Promoting physical activity, supporting mental health, and addressing restrictive eating behaviors may help reduce the risk of food addiction. Future research should adopt longitudinal designs and include detailed dietary assessments to better understand causal relationships and the impact of specific food choices on addictive eating patterns.

## Data Availability

The data used and/or analyzed during the current study are available from the corresponding author(s) upon reasonable request.
